# Experimental Study on the Effects of Thermal Cycling and Ultraviolet Irradiation on Stable Characteristics of Carbon Fiber/Bismaleimide Polymer Composite Shells

**DOI:** 10.3390/ma18091942

**Published:** 2025-04-24

**Authors:** Zheng Zhang, Xinyue Ji, Min Sun, Libin Xiong, Guang Zhang, Wenjie Ding, Jiquan Li, Shaofei Jiang

**Affiliations:** 1 College of Mechanical Engineering, Zhejiang University of Technology, Hangzhou 310023, China; zzhangm@zjut.edu.cn (Z.Z.); jixywork@163.com (X.J.); libin.xiong@leadgp.com (L.X.); guangzhang@zjut.edu.cn (G.Z.); dingwenjie_1978@126.com (W.D.); lijq@zjut.edu.cn (J.L.); jsf75@zjut.edu.cn (S.J.); 2 Key Laboratory of Special Purpose Equipment and Advanced Processing Technology, Ministry of Education and Zhejiang Province, Zhejiang University of Technology, Hangzhou 310023, China; 3 Institute of Advanced Structure Technology, Beijing Institute Technology, Beijing 100081, China

**Keywords:** bistable composite shells, carbon fiber, bismaleimide resin, epoxy resin, aging

## Abstract

Carbon fiber/epoxy composites are widely used in aerospace due to their strength and ease of fab weatherrication, but they suffer from glass transition at high temperatures. In contrast, carbon fiber/bismaleimide composites exhibit superior thermal stability, irradiation resistance, and toughness. This study investigates the effects of thermal cycling and UV irradiation on carbon fiber/bismaleimide composite shells. Anti-symmetric shells with varying ply angles were tested under two-point tensile loading. Results show that these shells maintain stability between −70 °C and 180 °C, outperforming epoxy-based composites. This research offers valuable insights for aerospace applications of such bistable shells under thermal and UV aging conditions.

## 1. Introduction

Carbon fiber/epoxy polymer (CFEP) composites are extensively utilized in aerospace applications due to their low specific gravity, high strength, high stiffness, and ease of fabrication [1]. Bistable shells made of CFEP composite are widely employed in aerospace applications as a novel space-deployable shell structure featuring two stable configurations [2,3,4]. Due to their smaller driving forces and larger deformations, bistable composite shells have been the subject of many studies that have explored the effects of temperature and humidity on bistable composite shells made of CFEP, including their fracture behavior, curvature changes, and microstructural characteristics [5,6,7,8,9,10,11,12,13]. Zhang et al. [14,15,16,17,18,19] investigated snap behaviors, curvature change, and microstructural analysis of bistable cylindrical shells made of CFEP composite following thermal treatment. Rio et al. [20] investigated the mechanical behavior of bistable shells made of CFEP composite under low-velocity impact conditions, testing temperatures ranging from 20 °C to −150 °C. However, CFEP composites tend to undergo glass transition when exposed to high temperatures, which can compromise their mechanical properties and the stability of the shell’s configurations [21,22,23,24,25,26]. This limitation has driven the search for alternative composite materials that can withstand the extreme conditions encountered in aerospace applications.

Carbon fiber/bismaleimide polymer (CFBP) composites are widely applied in deployable structures in aerospace applications due to their high heat resistance [27,28,29], robust irradiation resistance [30], and impressive corrosion resistance [31,32,33]. Bistable shells made of CFBP composites exhibit excellent stability of the shell’s configuration under thermal cycling and ultraviolet irradiation in aerospace applications, due to the high modulus and low thermal expansion of the CFBP composites [34,35]. Sun et al. [36] investigated the correlation between the heat-resistant interface and the thermal oxidation aging properties of CFBP composites. Shimokawa et al. [33] studied the impact of thermal cycling on microcrack formation and compressive strength in CFBP composites from −54 °C to 100 °C. Zhan et al. [37] evaluated the mechanical properties of bistable shells made of CFBP composite with a central opening specimen under ultraviolet irradiation by static tensile and compression tests. Although there have been many studies on the influence of CFBP composites [27,38,39,40], there are still limitations in the study of the influence of bistable shells made of CFBP composite under thermal and ultraviolet aging in aerospace application. Therefore, it is essential to investigate the effects of thermal cycling and ultraviolet irradiation on the stable characteristics of bistable shells made of CFBP composite to ensure their reliability and performance in aerospace applications.

The paper is organized as follows: Section 2 describes the fabrication of tensile specimens and bistable composite shells with orthogonal laminates. The mechanical properties and heat resistance of bismaleimide resin and epoxy resin were obtained. In Section 3, the stability of stable configurations and the snap behaviors under pretreatments of different extreme environments were measured, which included high-temperature aging, low-temperature aging, thermal cycling aging, and ultraviolet irradiation aging. The concluding remarks are presented in Section 4.

## 2. Materials and Methods

### 2.1. Fabrication of Resin Specimens and Bistable Shells

**Figure 1:**
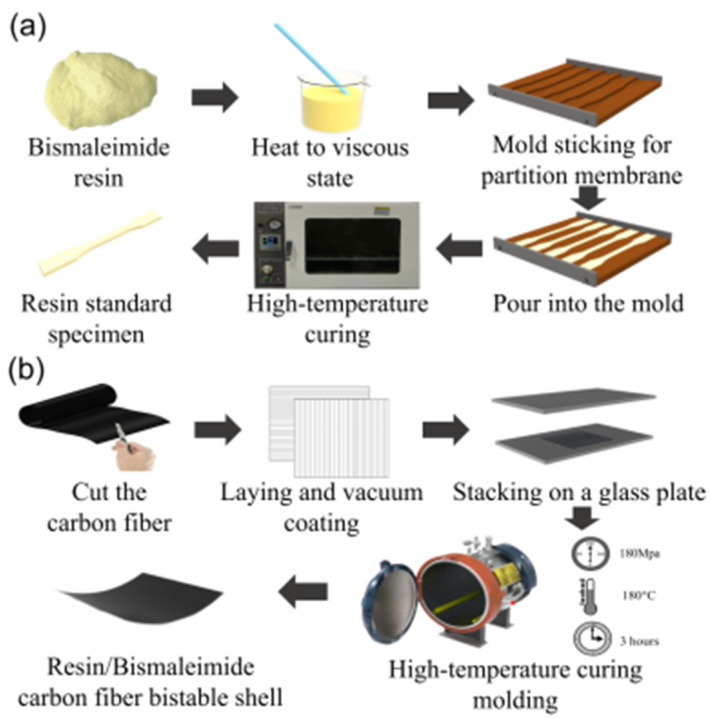
Fabrication of bistable shells made of CFBP and CFEP composites: (**a**) resin specimens, (**b**) bistable shells.

To measure the stable characteristics of bistable shells made of CFBP composite, tensile specimens of resins and bistable shells of carbon fiber/resin composites were fabricated according to the fabrication procedure in Figure 1. QY8911 bismaleimide resin was selected for its superior thermal stability and toughness [41]. HH2028 epoxy resin is recognized for its exceptional adhesion, chemical stability, mechanical strength, and ease of processing [42]. T700 carbon fiber was selected for its high strength and modulus, extended fatigue life, and enhanced performance under diverse loading conditions, which are superior to T300 carbon fiber [43]. QY8911 bismaleimide resin, HH2028 epoxy resin, and T700 carbon fiber were supplied by Dongguan Henghai Composite Materials Technology Co., Ltd. (Dongguan City, China). Tensile specimens of both epoxy and bismaleimide resins were fabricated through a four-step procedure, as shown in Figure 1a: (1) Pour stirred resin and curing agent into the beaker and put it into a vacuum drying oven set at 70 °C for three hours until it reaches a viscous state. (2) Attach the partition membrane to the mold and pour the resin. (3) Put the resin-filled mold into the vacuum drying oven for curing according to the curing temperature and time of the resin. (4) Remove the side baffles and drop out the resin tensile specimen.

Moreover, bistable shells made of CFBP and CFEP composites were fabricated, as shown in Figure 1b. The three fabrication steps of the bistable shells made of CFBP and CFEP composites are as follows: (1) Cut the T700/QY8911 prepreg into 10 cm × 10 cm-sized pieces and lay the layers on a plate by orthogonal ply. (2) Place the orthogonally laminated shells into the autoclave at a pressure of 0.6 MPa and a temperature of 180 °C for 3 h. (3) Cool down to room temperature and take out the orthogonal bistable composite shells.

### 2.2. Tensile Mechanical Properties and Thermal Resistance of Resins

**Figure 2:**
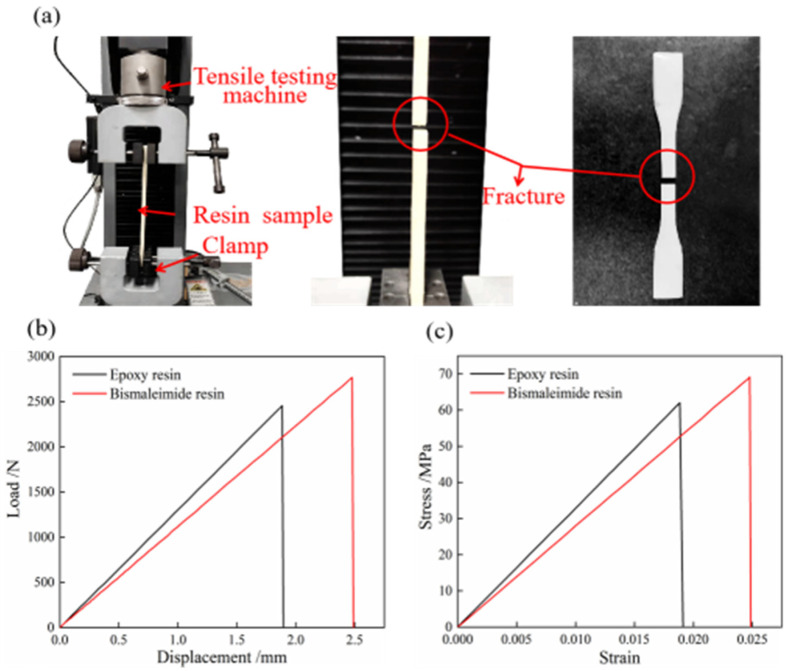
Tensile mechanical properties of epoxy and bismaleimide resins: (**a**) fracture process, (**b**) load–displacement curve, (**c**) stress–strain curve.

It is crucial to investigate the tensile mechanical properties and thermal resistance of epoxy and bismaleimide resins to ensure their suitability for aerospace applications. Figure 2 illustrates the tensile mechanical properties of epoxy and bismaleimide resins. The mechanical and heat resistance properties can be obtained using a tensile testing machine (Instron Legend 2345, Norwood, MA, USA) with a 5 kN force sensor and a dynamic mechanical analyzer (DMA 2980, New Castle, DE, USA), respectively. As shown in Figure 2a, the two ends of the bismaleimide resin standard specimen were fixed by clamps, and the loading speed of the stretching process was adjusted to 10 mm/min. The resin specimens broke with a smooth fracture after the loading process reached 5 s. After outputting real-time data processing, the load–displacement curve is shown in Figure 2b and the stress–strain curve is shown in Figure 2c.

To further study the mechanical properties of epoxy and bismaleimide resins, it is necessary to calculate the three key mechanical properties: longitudinal tensile strength, elastic modulus, and elongation. These properties were used to characterize the maximum strength range, rigidity, and toughness of the epoxy and bismaleimide resins, respectively. Moreover, the three properties were calculated according to the international standard (ISO 527-2:2012) [44]. The calculation equations of tensile strength, elastic modulus, and elongation are shown in Equations (1)–(3).

(1) $$\delta_{t}=\frac{P_{b}}{b\times h}$$

(2) $$E=\frac{L_{0}\times \Delta P}{b\times h\times \Delta L}$$

(3) $$\varepsilon_{t}=\frac{\Delta L_{b}}{\Delta L_{0}}\times 100$$

where *δ*_t_ represents the longitudinal tensile strength of the resin specimen, and *P*_b_ denotes the maximum load at break. *b* and *h* represent the width and thickness of the narrow range for the resin specimen, respectively. *E* and *L*_0_ represent the longitudinal tensile elastic modulus and the gauge length of the narrow section of the resin, respectively. *ΔP* indicates the load increment in the linear stage of the load–displacement curve, and *ΔL* represents the corresponding deformation displacement increment of the gauge length *L*_0_. *ε*_t_ refers to the elongation at break, while *ΔL*_b_ signifies the fracture deformation elongation of the resin specimen within the range of gauge length *L*_0_.

The values of tensile strength, elastic modulus, and elongation for the epoxy and bismaleimide resins, calculated using Equations (1)–(3), are detailed in Table 1. It can be seen that the tensile strength and elongation at break of the bismaleimide resin are higher than those of the epoxy resin, but the epoxy resin has a higher elastic modulus. Due to the repeated snap processes, bistable shells need to exhibit characteristics of high strength and high toughness. Therefore, the resin matrix as a composite material with a bistable structure is required to possess excellent tensile strength. Comparing the mechanical properties of the two resin matrices comprehensively, the bismaleimide resin as the matrix of the bistable composite structure has better mechanical behavior.

Resins utilized in aerospace applications must possess not only superior mechanical properties but also excellent heat resistance to endure the high temperature conditions [45]. Since high temperatures can significantly impair the working performance of resins, it is essential to compare the heat resistance of epoxy and bismaleimide resins [46]. The DMA was employed to ascertain the real-time dependence of storage modulus, loss modulus, and loss factor on experimental temperature, following a three-step procedure: (1) Cut the cured resin into a rectangular block specimen, with a size of 50 mm × 12.4 mm × 2.3 mm. (2) Install the fixture for fixing the resin specimens, and perform the quality correction and compliance correction. (3) Obtain the real-time temperature loss factor through DMA.

**Figure 3:**
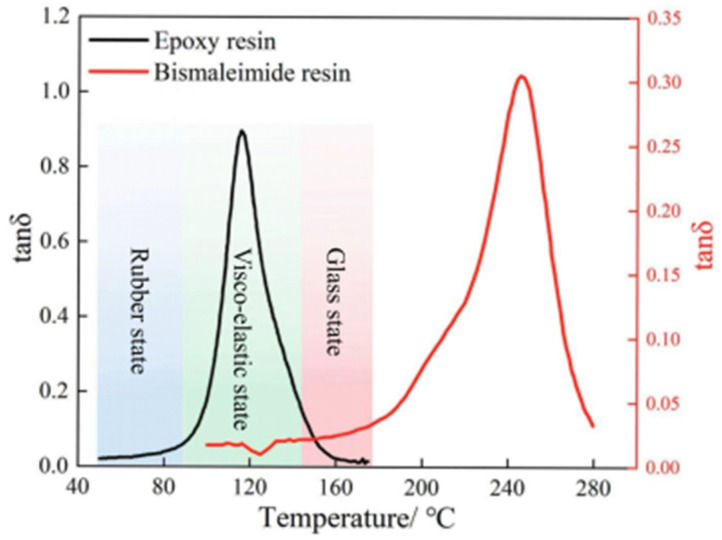
Glass transition temperature of epoxy and bismaleimide resins.

The real-time correlation data of the epoxy and bismaleimide resins between the loss factor tanδ and temperature can be obtained through dynamic mechanical analysis, and the results are presented in Figure 3. The trends of the loss factors of the epoxy and bismaleimide resins are similar: all increase first, then decrease as temperature rises. Meanwhile, the resin matrices transform from a rubber state to a viscoelastic state and finally to a glass state as the temperature rises. Moreover, the temperature corresponding to the peak loss factor is the glass transition temperature of the resin, and the glass transition temperatures of epoxy and bismaleimide resins are 115 °C and 247 °C, respectively. Comparing the glass transition temperature of epoxy and bismaleimide resins, epoxy resin has poor heat resistance. Therefore, the bismaleimide resin, with high heat resistance, is more suitable for high-temperature environments in aerospace applications.

### 2.3. Stable Characteristics of Bistable Shells

Bistable composite shells in aerospace applications are subjected to thermal and ultraviolet irradiation environments, emphasizing the importance of temperature and ultraviolet simulation [47]. The pretreatments of bistable shells encompass simulating high-temperature aging, low-temperature aging, thermal cycling aging, and ultraviolet irradiation aging environments to assess the impact on the stable characteristics of carbon fiber composite shells. A programmable constant temperature and humidity test chamber were utilized to simulate high-temperature aging, low-temperature aging, and thermal cycle aging environments and an ultraviolet accelerated aging test chamber was employed to simulate the ultraviolet irradiation aging environment. Concurrently, a tensile testing machine (Instron Legend 2345, Norwood, MA, USA) and a 3D scanner (FreeScan Trio, Beijing, China) were used to measure snap loads and the stable configurations of bistable shells after aging.

To simulate the aging process of bistable composite shells, the programmable constant temperature and humidity test chamber (Thermo Fisher Scientific, Waltham, MA, USA) were set to a temperature range of 150 °C for high-temperature aging and −70 °C for low-temperature aging. The aging times were set to 0 h, 10 h, 20 h, and 40 h. In the ultraviolet accelerated aging test chamber, the input current of the irradiation lamp was set to 300 mA, corresponding to an irradiation intensity of 1.2 W/m^2^. The aging times were set to 0 h, 10 h, 20 h, and 30 h. For the high- and low-temperature cycling aging test, the temperature was set to range from 150 °C to −70 °C, with cycle numbers set to 5, 10, and 15 with a 20 min interval.

**Figure 4:**
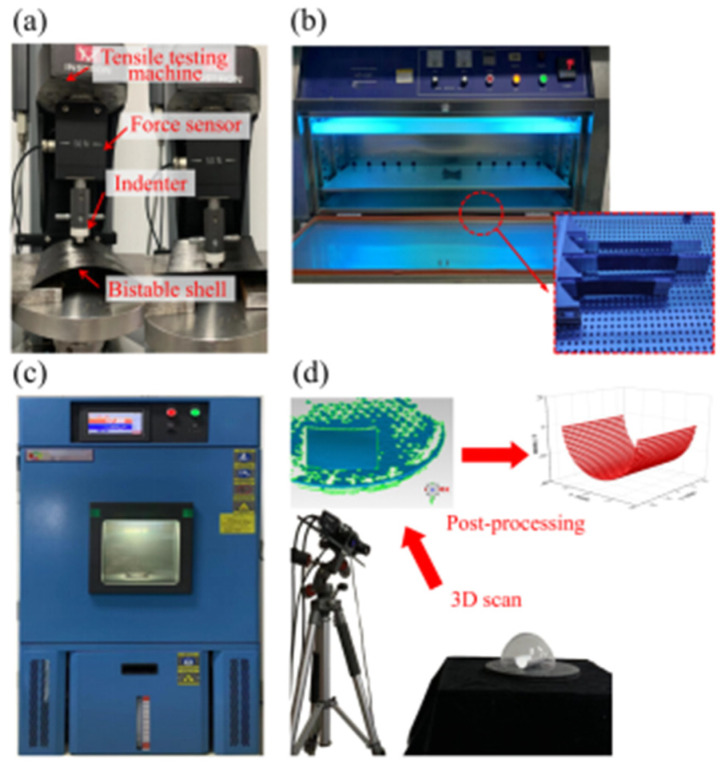
Thermal and ultraviolet irradiation aging of bistable shells: (**a**) tensile testing machine, (**b**) constant-temperature and humidity test chamber, (**c**) ultraviolet-accelerated aging test chamber, (**d**) 3D scanner.

Due to the high susceptibility of bistable composite shells used in aerospace applications, such as antenna substrate structures, to thermal and ultraviolet environments, it becomes necessary to imitate these environmental conditions. The experimental processes were used to imitate the thermal and ultraviolet environments and to obtain snap loads, and the curvatures of stable configuration are presented in Figure 4. The 3D scanner and tensile testing machines were utilized to obtain the stable configurations and snap behaviors of the two stable states, including curvatures and snap loads, through a four-step procedure: (1) Prepare the bistable composite shells using bismaleimide and epoxy resin matrices. (2) Put them into the constant-temperature, humidity test chamber, and the ultraviolet-accelerated aging test chamber, respectively. (3) Carry out aging treatments according to the above setting values. (4) Measure the curvatures of stable configurations and snap loads of the specimens.

## 3. Results

### 3.1. High-Temperature Aging

Since bistable composite shells have to operate in high-temperature environments reaching up to 150 °C, further investigation is required to understand the long-term effects of ambient temperature aging on these shells. Therefore, it is essential to evaluate the curvatures of stable configurations and the snap loads of bistable composite shells under high-temperature aging [48].

**Figure 5:**
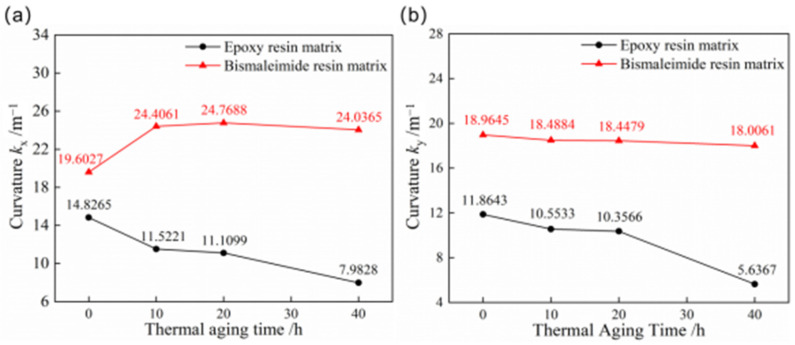
Curvatures of bistable shells made of CFBP and CFEP composites after high-temperature aging: (**a**) first stable state, (**b**) second stable state.

The curves of curvatures of the first and second stable configuration for bistable shells made of CFBP and CFEP composites after high-temperature aging are depicted in Figure 5. The stable configuration curvatures of bistable shells made of CFEP composite are significantly influenced by high temperatures, whereas the curvatures of bistable shells made of CFBP composite exhibit minimal change. Moreover, as the aging time progresses, the curvature of the first stable configuration of the bistable shell made of CFBP composite initially increases and then declines, whereas the curvature of the second stable configuration gradually decreases. The changes in curvature for the bistable shell made of CFBP composite’s first stable configuration, which initially increases and then decreases, may be attributed to the stress accumulation in the laminated shell due to the secondary curing of bismaleimide resin. This secondary curing effect accounts for the initial rise in curvature of the first stable state. However, the curvature of the second stable state, which possesses higher potential energy, gradually decreases due to thermal stress at elevated temperatures. The experimental temperature of 150 °C is 115 °C above the glass transition temperature of the epoxy resin matrix, leading to rapid resin decomposition and a consequent decrease in the curvatures of both stable states for the bistable shell made of CFEP composite. Conversely, the bistable shell made of CFBP composite shows greater stability in maintaining its configuration after 40 h of high-temperature aging, surpassing the bistable shell made of CFEP composite.

**Figure 6:**
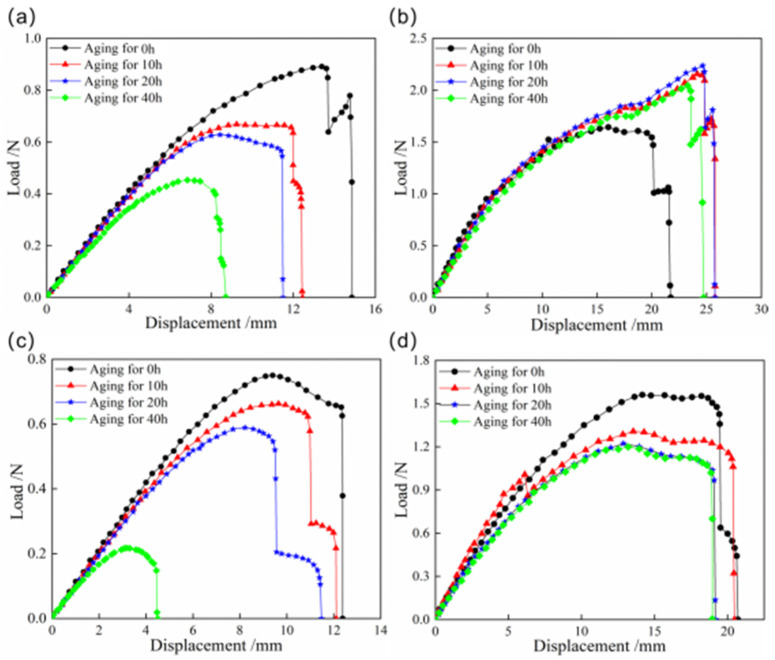
Load–displacement curves of snap processes of bistable shells made of CFBP and CFEP composites after high-temperature aging: (**a**) snap-through process of epoxy resin matrix, (**b**) snap-through process of bismaleimide resin matrix, (**c**) snap-back process of epoxy resin matrix, (**d**) snap-back process of bismaleimide resin matrix.

To further investigate the snap behaviors of bistable shells made of CFBP composite after high-temperature aging, Figure 6 presents the load–displacement curves of the snap processes for both bistable shells made of CFBP and CFEP composites after high-temperature aging. It is observed that the snap-through load of the bistable shell made of CFBP composite increases with aging time, whereas the snap-back load decreases. This trend is attributed to the secondary curing of the resin, which enhances the mechanical properties of the bistable shell made of CFBP composite. Conversely, the high-temperature aging process reduces the thermal stress within the second stable state of higher potential energy, leading to a corresponding decrease in snap-back loads over time. For the bistable shell made of CFEP composite, both snap-through and snap-back loads decrease with increasing aging time, due to a rapid decline in mechanical properties under prolonged exposure to high temperatures beyond the glass transition temperature. Consequently, after 40 h of high-temperature aging, the bistable shell made of CFBP composite exhibits superior mechanical properties compared to the bistable shell made of CFEP composite.

### 3.2. Low-Temperature Aging

**Figure 7:**
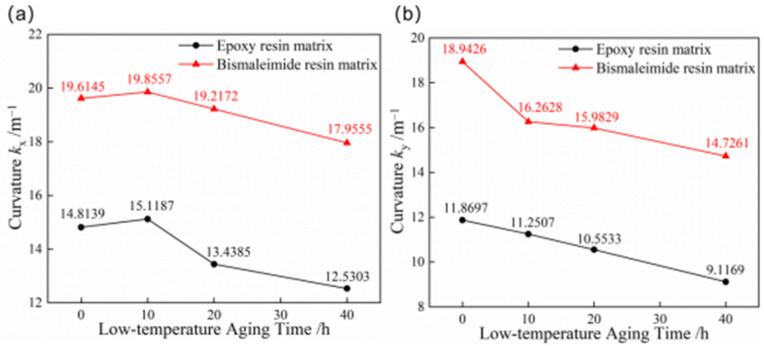
Curvatures of bistable shells made of CFBP and CFEP composites after low-temperature aging: (**a**) first stable state, (**b**) second stable state.

Bistable composite shells are subjected to low-temperature conditions in aerospace applications, making it necessary to study the effects of low-temperature aging on bistable composite shells. Figure 7 compares the curvatures of the first and second stable states of bistable shells made of CFBP and CFEP composites after such treatment. The curvatures of the first stable configurations for both bistable shells made of CFBP and CFEP composites increase after 10 h of low-temperature aging but decrease with further aging. This is due to an increase in stress differences between the x and y directions of the bistable composite shell, which initially leads to higher curvatures. However, the curvatures of the second configuration for both the epoxy and bistable shells made of CFBP composite continuously decrease during low-temperature aging, possibly due to a reduction in the crosslinking degree of the resin and fiber over time, resulting in decreased stable configuration curvatures. Moreover, the bistable shell made of CFBP composite exhibits better stability when comparing curvature changes under low-temperature aging for both stable states.

**Figure 8:**
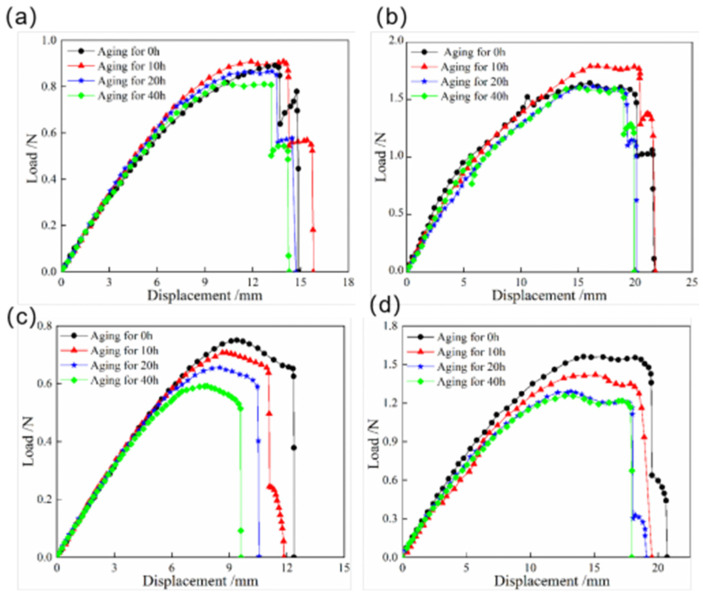
Load–displacement curves of snap processes of bistable shells made of CFBP and CFEP composites after low-temperature aging: (**a**) snap-through process of epoxy resin matrix, (**b**) snap-through process of bismaleimide resin matrix, (**c**) snap-back process of epoxy resin matrix, (**d**) snap-back process of bismaleimide resin matrix.

The load–displacement curves of the snap processes for bistable shells made of CFBP and CFEP composites under low-temperature aging are shown in Figure 8. These curves show a similar trend for both resin bistable composite shells, with an initial increase followed by a decrease during the low-temperature aging process. This behavior is attributed to the hardening of the resin matrix and the increased interfacial reinforcement between the carbon fiber and the resin matrix after 10 h of low-temperature aging, which initially leads to an increase in the snap load. After low-temperature aging, the reduction in snap load for the bistable shell made of CFEP composite is less than that for the bistable shell made of CFBP composite. However, the snap load of the bistable shell made of CFBP composite is approximately twice that of the CFEP shell. Consequently, the bistable shell made of CFBP composite exhibits superior performance in terms of stable state after low-temperature aging.

### 3.3. Thermocycling Aging

**Figure 9:**
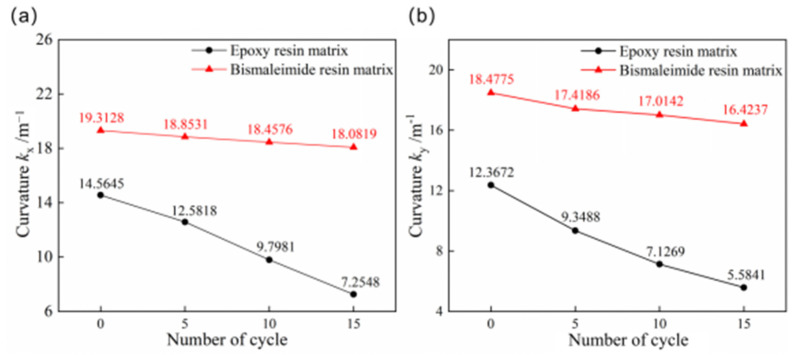
Curvatures of bistable shells made of CFBP and CFEP composites after thermocycling aging: (**a**) first stable state, (**b**) second stable state.

It is necessary to conduct thermal cycling experiments on bistable structures due to the significant diurnal temperature variations in aerospace applications. Figure 9 illustrates the comparison of the first and second stable state curvatures of bistable shells made of CFBP and CFEP composites after thermocycling aging. The curvatures of both the first and second stable states for bistable shells made of CFBP and CFEP composites decrease with increasing thermal cycle aging times, with bistable shells made of CFEP composite showing a greater reduction. Consequently, the curvatures of stable configuration for bistable shells made of CFEP composite are more significantly affected by thermocycling aging compared to bistable shells made of CFBP composite.

**Figure 10:**
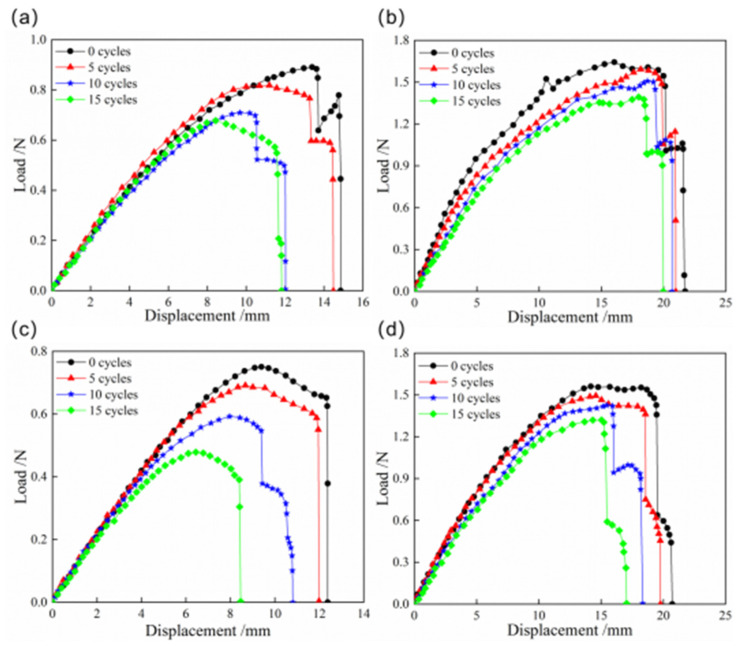
Load–displacement curves of snap processes of bistable shells made of CFBP and CFEP composites after thermocycling aging: (**a**) snap-through process of epoxy resin matrix, (**b**) snap-through process of bismaleimide resin matrix, (**c**) snap-back process of epoxy resin matrix, (**d**) snap-back process of bismaleimide resin matrix.

The load–displacement curves of snap processes for CFEP and bistable shells made of CFBP composite after thermocycling aging are indicated in Figure 10. The snap loads of both bistable shells made of CFBP and CFEP composites decrease with an increasing number of cycles. This reduction is attributed to the degradation of the mechanical properties and the alleviation of residual stress due to the fluctuation between high and low temperatures. After 15 cycles of thermocycling aging, the snap loads of the bistable shell made of CFEP composite decrease by approximately 30% and 40%, respectively, compared to the shells with no thermocycling aging. However, the snap loads of the bistable shell made of CFBP composite only reduce by 15%. Additionally, oscillations in the load–displacement curve during the later stages are observed, attributed to buckling-induced local stable state transitions within the shell. Despite thermal cycling, the bistable shell made of CFBP composite retains its superior mechanical properties.

### 3.4. Ultraviolet Irradiation Aging

**Figure 11:**
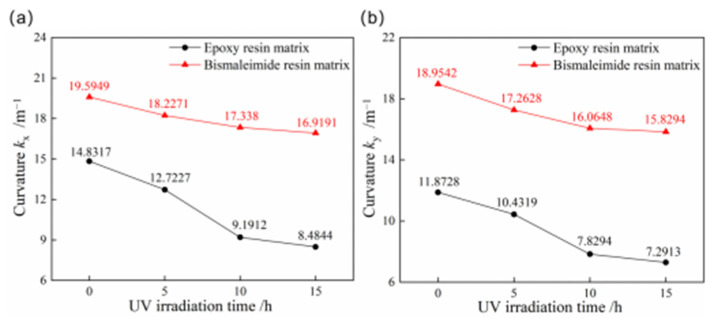
Curvatures of bistable shells made of CFBP and CFEP composites after ultraviolet irradiation aging: (**a**) first stable state, (**b**) second stable state.

Given the exposure to ultraviolet irradiation in aerospace applications, it is essential to investigate its effects on the stable characteristics of bistable composite shells. Figure 11 compares the curvatures of the first and second stable configurations for bistable shells made of CFBP and CFEP composites after ultraviolet irradiation aging. With increased ultraviolet irradiation aging time, both the curvatures of stable configuration for bistable shells made of CFBP and CFEP composites decrease, with the epoxy resin showing a more significant reduction. After 15 h of ultraviolet irradiation aging, the curvatures of the first and second stable configurations of bistable shells made of CFBP composite decreased by only 13.65% and 16.49%, respectively, whereas those of the bistable shells made of CFEP composite decreased by 42.80% and 38.58%. Therefore, the bistable shell made of CFBP composite can maintain better stability of configuration under ultraviolet irradiation of 1.2 W/m^2^ intensity.

**Figure 12:**
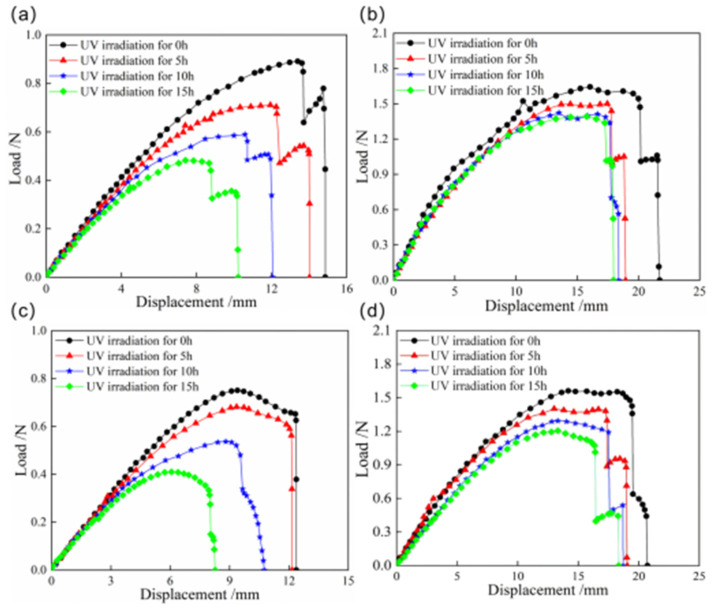
Load–displacement curves of snap processes of bistable shells made of CFBP and CFEP composites after ultraviolet irradiation aging: (**a**) snap-through process of epoxy resin matrix, (**b**) snap-through process of bismaleimide resin matrix, (**c**) snap-back process of epoxy resin matrix, (**d**) snap-back process of bismaleimide resin matrix.

The load–displacement curves of snap processes for bistable shells made of CFBP and CFEP composites after ultraviolet irradiation aging are presented in Figure 12. The snap loads of both bistable shells made of CFBP and CFEP composites decrease with increasing ultraviolet irradiation time. The bistable shell made of CFBP composite exhibits a smaller reduction in snap-through and snap-back loads compared to the bistable shells made of CFEP composite. Specifically, the snap-through and snap-back loads of bistable shells made of CFBP composite decrease by about 15%, whereas those of the bistable shells made of CFEP composite decrease by about 50%. Additionally, the maximum displacements during the snap processes of the bistable shells made of CFEP composite also decrease with longer aging times, with a reduction range significantly greater than that of the bistable shell made of CFBP composite. Consequently, the bistable shell made of CFBP composite maintains better mechanical properties of snap processes under ultraviolet irradiation aging, without a rapid decline.

## 4. Conclusions

The tensile mechanical properties and heat resistance of epoxy resin and bismaleimide resin were compared and bistable composite shells were prepared in this paper. Bismaleimide resin has higher tensile strength and elongation at fracture, but a smaller elastic modulus compared with epoxy resin. Meanwhile, the glass transition temperature of bismaleimide resin is much higher than that of epoxy resin. The aging treatment of bistable shells made of CFBP and CFEP composites was conducted under high-temperature, low-temperature, thermal cycle and ultraviolet irradiation environments. The results show that the curvatures of the stable configuration and snap loads of the bistable shell made from CFBP composite decrease by approximately 5% and 7%, respectively, while those of the bistable shell made from CFEP composite decrease by 8% and 16% after high-temperature aging for 40 h. Moreover, the curvatures of stable configuration and snap loads of the bistable shell made of CFEP composite decreased greatly under low-temperature, thermal cycle, and ultraviolet irradiation aging, and the reduction ranges of both are about 20~35% and 30~40%, respectively. Although the curvatures of stable configuration and snap loads of bistable shells made of CFBP and CFEP composites also changed under various environments, the bistable shell made of CFBP composite shows better stability of stable configurations. Furthermore, the light weight of the bistable shells makes them suitable for deployable structures in aerospace applications such as deployable cylindrical bistable shell antennas and deployable solar array bistable booms.

## Figures and Tables

**Table 1:** Mechanical properties of epoxy and bismaleimide resins.

Resin Type	Tensile Strength/MPa	Elastic Modulus/MPa	Elongation at Break/%
Epoxy	61.39	3295	3.16
Bismaleimide	69.18	2792	4.15

## Data Availability

The original contributions presented in this study are included in the article. Further inquiries can be directed to the corresponding author.
